# Abnormal Trafficking of Endogenously Expressed *BMPR2* Mutant Allelic Products in Patients with Heritable Pulmonary Arterial Hypertension

**DOI:** 10.1371/journal.pone.0080319

**Published:** 2013-11-05

**Authors:** Andrea L. Frump, Jonathan W. Lowery, Rizwan Hamid, Eric D. Austin, Mark de Caestecker

**Affiliations:** 1 Department of Cell and Developmental Biology, Vanderbilt University Medical Center, Nashville, Tennessee, United States of America; 2 Department of Developmental Biology, Harvard University School of Dental Medicine, Boston, Massachusetts, United States of America; 3 Department of Pediatrics, Division of Molecular Genetics and Genomic Medicine, Vanderbilt University Medical Center, Nashville, Tennessee, United States of America; 4 Department of Pediatrics, Division of Pediatric Pulmonary Medicine, Vanderbilt University Medical Center, Nashville, Tennessee, United States of America; 5 Department of Medicine, Vanderbilt University Medical Center, Nashville, Tennessee, United States of America; University of Pittsburgh School of Medicine, United States of America

## Abstract

More than 200 heterozygous mutations in the type 2 BMP receptor gene, *BMPR2*, have been identified in patients with Heritable Pulmonary Arterial Hypertension (HPAH). More severe clinical outcomes occur in patients with *BMPR2* mutations by-passing nonsense-mediated mRNA decay (NMD negative mutations). These comprise 40% of HPAH mutations and are predicted to express *BMPR2* mutant products. However expression of endogenous NMD negative *BMPR2* mutant products and their effect on protein trafficking and signaling function have never been described. Here, we characterize the expression and trafficking of an HPAH-associated NMD negative *BMPR2* mutation that results in an in-frame deletion of *BMPR2 EXON2* (*BMPR2ΔEx2*) in HPAH patient-derived lymphocytes and in pulmonary endothelial cells (PECs) from mice carrying the same in-frame deletion of *Exon 2* (*Bmpr2* ^*ΔEx2/+*^ mice). The endogenous *BMPR2ΔEx2* mutant product does not reach the cell surface and is retained in the endoplasmic reticulum. Moreover, chemical chaperones 4-PBA and TUDCA partially restore cell surface expression of *Bmpr2ΔEx2* in PECs, suggesting that the mutant product is mis-folded. We also show that PECs from *Bmpr2* ^*ΔEx2/+*^ mice have defects in the BMP-induced Smad1/5/8 and Id1 signaling axis, and that addition of chemical chaperones restores expression of the Smad1/5/8 target Id1. These data indicate that the endogenous NMD negative *BMPRΔEx2* mutant product is expressed but has a folding defect resulting in ER retention. Partial correction of this folding defect and restoration of defective BMP signaling using chemical chaperones suggests that protein-folding agents could be used therapeutically in patients with these NMD negative *BMPR2* mutations.

## Introduction

Despite modern vasodilator treatments, patients with Pulmonary Arterial Hypertension (PAH) only have a 50% 5-year survival[1]. For this reason there was considerable interest in the discovery that patients with a rare form of autosomal dominant, Heritable Pulmonary Arterial Hypertension (HPAH) carry mutations in the *BMPR2* (bone morphogenetic protein receptor 2) gene [2,3]. BMPR2 is one of three Type 2 BMP pathway receptors. Once stimulated by BMP ligands BMPR2 forms hetero-tetrameric complexes with Type I receptors at the cell surface (reviewed in 4,5). These receptor complexes then activate the canonical SMAD 1/5/8 pathway, which in turn leads to the transcriptional regulation of target genes such as *ID1*[6]. Several SMAD-independent pathways including ERK1/2 and p38 MAPK, PI3K and PKC are also activated downstream of BMPR2, but unlike canonical SMAD signaling, activation of these pathways is often cell type and context dependent [5,7]. Although the mechanism by which defects in the BMP signaling contribute to HPAH remains elusive, BMP-signaling defects associated with mutations in *BMPR2* have been implicated in abnormal pulmonary vascular cell proliferation, remodeling and vascular tone [8-14]. These diverse roles in regulating the pulmonary vasculature and the defects in this pathway detected in patients with HPAH, suggest that strategies to correct BMP signaling defects may have long-term disease modifying effects. To this end, approaches have been developed to enhance expression of the wild type *BMPR2* allelic product in HPAH patients [15]. In addition, drugs that promote read-through of pre-termination codons associated with *BMPR2* mutation that result in non-sense mediated decay of the mutant mRNA product, also increase expression of functional BMPR2 mutant products in cells from patients with HPAH [16,17]. In these studies we take an alternative approach to correct BMP signaling defects by correcting abnormal trafficking of a *BMPR2* mutant protein product. 

Over 200 unique mutation sites have been identified throughout the open reading frame of the *BMPR2* gene in HPAH patients [18,19]. The majority of these mutations are non-sense or frame-shift mutations, leading to degradation of an unstable mRNA by nonsense mediated mRNA decay (NMD positive mutation), ultimately leading to haploinsufficiency [20]. However, 40% of HPAH-associated *BMPR2* mutations are mis-sense or in-frame deletion mutations, predicted to produce stable mRNA transcripts and express mutant protein products. Clinical data indicate that HPAH patients with these mutations have a more severe form of HPAH with reduced time until lung transplant and an earlier age of diagnosis[20], suggesting that these mutant protein products are expressed and may have dominant negative effects on BMPR2 function. Previous studies have characterized NMD negative *BMPR2* mutations using heterologous over-expression systems. These data suggest that HPAH-associated missense mutations in the ligand binding and kinase domains of BMPR2 are unable to traffic to the cell surface [21,22], and that trafficking and signaling can be restored using chemical chaperones [23]. These findings suggest that chemical chaperones, which correct folding and restore function of mutant protein products in a variety of heritable diseases including cystic fibrosis [24,25], might be used as disease modifying agents by restoring signaling function to some NMD negative *BMPR2* mutations in patients with HPAH. However there is no documented evidence that NMD negative *BMPR2* mutant proteins products are actually expressed endogenously in patients with HPAH, and no data to indicate whether chemical chaperones restore function in pulmonary vascular cells. 

In these studies, we evaluate endogenous expression and intracellular trafficking of *BMPR2* mutant products in lymphocytes from an HPAH patient with an NMD negative *BMPR2* mutation expressing an in-frame deletion of *BMPR2* EXON *2*, and in pulmonary endothelial cells (PECs) from mice carrying the same mutation. We show that these mutant receptor products are expressed, abnormally trafficked to the cell surface and that these defects are corrected by treatment with chemical chaperones. 

## Materials and Methods

### Ethics statement

Mice used in endothelial cell studies were approved by the Vanderbilt University Institutional Animal Care and Use Committee (Dr. Ron Emeson, IACUC chair) under protocol number M/11/015 and were adherent with the National Institutes of Health guidelines for care and use of laboratory animals under Vanderbilt animal welfare assurance licence number A3227-01.

The Vanderbilt University Medical Center Institutional Review Boards approved use of control and HPAH patient-derived lymphocytes and participants gave informed written consent for use of their lymphocyte cultures for study (IRB #9401"Genetic and Environmental Pathogenesis of PPH" RFA-HL-04-019, Dr. James A.S. Muldowney, chair, Institutional Review Board Health Sciences Committee #3).

### Mouse lines

Endothelial cell lines were isolated from *Bmpr2* ^*ΔEx2/+*^ mice (mice described previously [26]). Studies were approved by the Vanderbilt University Institutional Animal Care and Use Committee (see above for ethics statement). Mice were backcrossed onto a C57Bl/6J background for more than 9 generations. Genotyping was performed by PCR from ear punch DNA using the following primers: Common forward CCATGCTCTTTTGAAGATGG; Wild type reverse: GTCCCCTTTTGATTTCTCCCA (producing a 1kB WT product); and mutant reverse: GGCCGCTTTTCTGGATTCATC (producing a 700bp mutant product). To create the ciEC lines, *Bmpr2* ^*ΔEx2/+*^ mice were bred with H-2Kb-tsA58 immorto mice (immorto mice described previously [27]). Genotyping was performed as described above with the additional PCR primers for the immorto mice: forward: AGCGCTTGTGTCGCCATTGTATTC and reverse: GTCACACCACAGAAGTAAGGTTCC, producing a 1kb band. 

### Chemicals and Reagents

Sulfo-NHS-LC-Biotin and Streptavidin agarose conjugated beads (Pierce/Thermo Scientific); Dio-Ac-LDL (Biomedical Technologies Inc.); Endo-H and PNGase-F glycosidases (New England Biolabs); Human recombinant BMP-2 (R&D Systems); chemical chaperones sodium phenylbutyrate (4-PBA) and Sodium taurourdeoxycholic acid (TUDCA) (Sigma-Aldrich). The following antibodies were used for western blots: mouse monoclonal anti-BMPR2 (clone 18, BD Biosciences) and anti-β-actin (Sigma-Aldrich); rabbit polyclonal anti-phospho-Smad1 (Ser463, Ser 465) /5(Ser 463, 465) /8(Ser 426, 428), anti-phospho-Akt(Ser 473), and anti-phospho-Erk1/2 (Thr 202, Tyr 204) (Cell Signaling). Affinity purified rabbit polyclonal anti-BMPR2 antibodies were generated in house by immunizing rabbits with keyhole limpet hemocyanin conjugated peptide ASQNQERLCAFKDP (ASQ). Secondary antibodies were anti-mouse horseradish peroxidase (HRP) and anti-rabbit-HRP from KPL and Cell Signaling, respectively.

### Cell Culture

Conditionally immortalized pulmonary endothelial cells (ciPECs) were isolated, passaged and maintained as previously described [8,28,29]. Briefly, cells were grown at 33°C in complete EGM-2MV media (LONZA) containing 10units/ml of interferon-γ (Peprotech). Prior to experimental use, interferon-γ was removed and cells shifted from 33°C to 37°C for 2 days. HPAH patient-derived and normal control immortalized lymphocytes were isolated as previously described [16,30]. Cells were passaged and maintained in 1640 RPMI media (Gibco/Life Technologies) containing 20% fetal bovine serum (FBS) (Sigma). 

### Isolation of Primary Pulmonary Endothelial Cells (PECs)

Mouse pulmonary vasculature was perfused with phosphate buffered saline (PBS) containing 2mM EDTA followed by 0.25% Trypsin-EDTA through the right ventricle. The lungs were excised, minced, placed in a 60mm dish with 0.25% Trypsin-EDTA and moved to a 37° degree incubator for 20-30 minutes. The lung block was removed and complete EGM-2MV containing 5% FBS was then added to the dish to neutralize trypsin. Cells were centrifuged and re-suspended in fresh complete EGM-2MV with normacin (Invivogen). The cell suspension was plated on 35mm dishes coated in 0.1% gelatin and grown for 2-3 days before media was replaced. The cells were identified as endothelial in origin based on morphology and Dio-AC-LDL staining. Functional studies were carried out in the cells by passage 2.

### Western Blotting

Cells were lysed in ice cold lysis buffer (LB) made up of 25mM HEPES, 150mM NaCl, 5mM EDTA, 1% Triton X-100, 10% glycerol containing proteinase inhibitor cocktail and phosphatase inhibitor cocktails 2 and 3 (Sigma). Protein concentration was measured using the DC Protein Assay (Bio-Rad). Western blots were performed as previously described [31]. All primary antibodies were used at a dilution of 1:1000 in 5% nonfat dry milk in TBST (25mM Tris, 1M NaCl, 1% Tween 20), excepting phospho-specific antibodies which were diluted 1:1000 in 5% BSA in TBST. Secondary antibodies were diluted 1:2000 in 5% nonfat dry milk in TBST. 

### Cell signaling and chemical chaperone treatment

For signaling studies, cells were serum starved in EBM-2 media (Lonza) supplemented with 0.1% bovine serum albumin (BSA) for 16 hours and then treated with 10ng/ml BMP2 for 4 hours. EBM-2 is the basal media used to make complete EGM-2MV without addition of supplements, growth factors and FBS. Chemical chaperones were added to confluent monolayers of cells. 4-PBA and TUDCA were dissolved in water and added to cells at the indicated concentrations 48 hours and 5 hours prior to biotinylation, respectively. For functional studies, primary PECs were treated with 4-PBA at 1mM for 48 hours. Cells were serum starved for 16 hours, as described above, and treated with 10ng/ml of BMP2 for 4 hours before lysis. 

### Glycosidase Sensitivity Assay

Cells were lysed in LB and immunoprecipitated using Protein G dynabeads (Life Technologies) conjugated to anti-BMPR2 antibody (Clone 18, BD) at room temperature for 15 minutes, per the manufacturer’s directions. Immunoprecipitates were washed 3 times with LB, denatured using de-glycosylation buffer and subjected to Endo-H or PNGase-F digestion for 3 hours in a 37° C before adding sample loading buffer, according to the manufacturer’s instructions (New England Biolabs). 

### Biotinylation of Cell Surface Proteins

Confluent monolayers were washed 2 times with ice-cold PBS and cell surface proteins labeled with 1mg/ml Sulfo-NHS-LC-Biotin in PBS for 30 minutes on ice and at 4°C. Cells were washed 3 times with ice cold PBS and biotin labeling quenched by incubating cells with 100mM glycine in PBS for 5 minutes on ice. After this, cells were carefully washed in ice cold PBS to remove any residual glycine. Cells were then lysed in LB and protein concentration determined. 750µg of lysate protein was incubated for 30 minutes with Streptavidin beads at 4°C, after which the beads were washed 3 times with LB before adding denaturing loading buffer and separating by SDS-PAGE. Supernatant leftover after the streptavidin pull-down was incubated with anti-BMPR2 antibody (Clone 18, BD) overnight at 4°C and then subjected to immunoprecipitation with Protein A/G Plus agarose beads (Santa-Cruz) for 30 minutes. Immunoprecipitates were washed with LB and proteins eluted by adding sample loading buffer. 

### Statistical Analysis

Statistical analyses were performed using one-way ANOVA with Multiple comparisons between groups and Bonferroni Comparison test correction post hoc with Graphpad Prism 5 software. Significance is indicated if p<0.05. 

## Results

### Characterization of the BMPR2ΔEx2 mutant protein product in HPAH patient-derived lymphocytes

**Figure 1:**
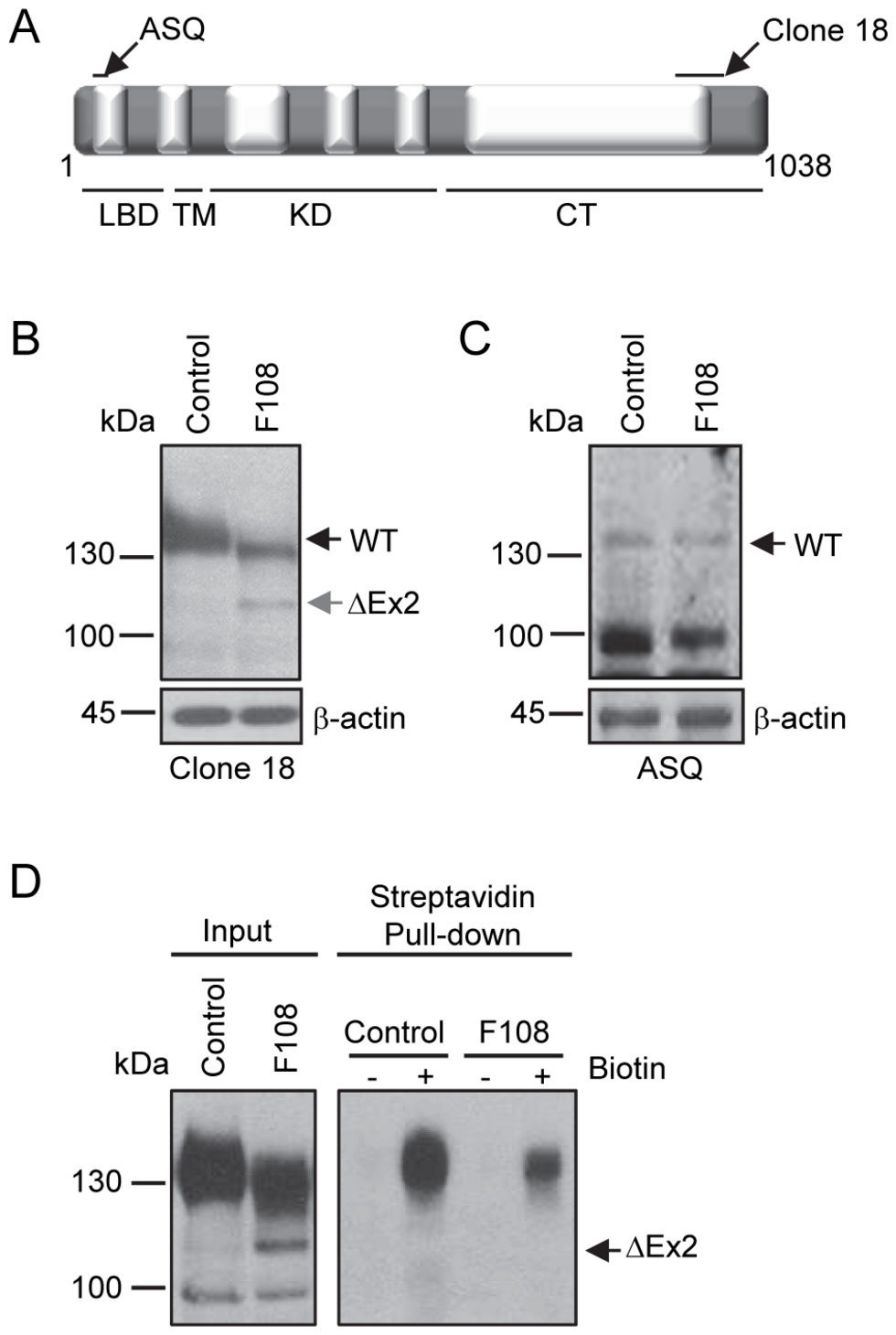
HPAH patient-derived lymphocytes express mutant BMPR2 products. A, Schematic of the BMPR2 protein. Areas recognized by the anti-BMPR2 antibodies clone 18 and ASQ are indicated by black arrows. Exons 1-13 are represented by alternating gray and white boxes, and numbers indicate corresponding amino acids. LBD, represents the ligand binding domain, TM the transmembrane domain, KD the kinase domain and CT the cytoplasmic tail. B, Detection of BMPR2 products in HPAH patient-derived lymphocytes, image representative of three experiments. Western blot using anti-BMPR2 antibody, Clone 18. A 130-145 kDa wild type BMPR2 product (WT) was detected in normal control and Family 108 (F108) HPAH patient-derived lymphoblasts. F108 cells expressed an additional 120 kDa BMPR2 mutant product (ΔEx2). C, Representative western blot from three experiments using the ASQ anti-BMPR2 antibody. The 130-145 kDa product was detected in both control and F108 lymphocytes, but the 120 kDa band was not detected. D, Cell surface expression of BMPR2 in HPAH patient-derived lymphocytes labeled with a membrane impermeable biotin. Experiment replicated three times. Left panel, input cell lysates before the streptavidin pull-down. Right panel, cell surface proteins detected in streptavidin pull-down by Western blot using Clone 18 anti-BMPR2 antibody. A 130-145 kDa wild type BMPR2 product was detected in control and F108 cultured lymphocytes in streptavidin pull-down but not 120kDa BMPR2ΔEx2 mutant product.

To determine if NMD negative *BMPR2* mutations are expressed and incorrectly trafficked to the cell surface in patients with HPAH, we evaluated BMPR2 protein expression in HPAH patient-derived lymphocytes [16,30]. Cultured immortalized lymphocytes were used because these cells are easily isolated and stored. Additionally, we have a repository of frozen, HPAH patient-derived cultured lymphocytes at Vanderbilt from participants in the Vanderbilt Prospective Pulmonary Hypertension Research Cohort study [16,19,30,32-35]. For these studies we evaluated BMPR2 expression in lymphocytes derived from an HPAH patient from Vanderbilt PAH Family 108 (F108) who carry a splice site mutation predicted to result in an in-frame deletion of *BMPR2 EXON2*, which encodes residues 26-82 of the 1038 full length BMPR2 protein[30]. If expressed, the mutant product, BMPR2ΔEx2, would be distinguishable from the wild type allelic product by a mobility shift on western blot resulting from deletion of 56 amino acids encoded by *EXON2*. We obtained lymphocytes from one normal control and one F108 HPAH patient. The anti-BMPR2 antibody, Clone 18, which is a mouse monoclonal antibody raised against a recombinant fragment (residues 803-996) from the cytoplasmic tail of human BMPR2 (Figure 1A), detected a strong 130-145 kDa wild type BMPR2 band in control and F108 HPAH patient lymphocytes (Figure 1B). An additional weaker 120 kDa band was detected in F108 HPAH, but not in normal control cells. To determine if the 120 kDa band was the predicted product resulting from in-frame deletion of *BMPR2 EXON2* in F108 HPAH lymphocytes, we generated an affinity purified rabbit polyclonal anti-BMPR2 antibody, ASQ, raised against the peptide sequence of the first 14 amino acids encoded by *BMPR2 EXON2* (Figure 1A). The 130-145 kDa wild type BMPR2 band was detected in control and F108 lymphocytes, but the 120 kDa BMPR2 band was not detected in F108 cells using this antibody (Figure 1C). These data indicate that lymphocytes from a F108 HPAH patient express a mutant BMPR2 product resulting from an in-frame deletion of *BMPR2 EXON2* (BMPR2ΔEx2). 

We next evaluated if the BMPR2ΔEx2 product was expressed at the cell surface. For this, cell surface proteins were labeled with a membrane impermeable biotin and detected by streptavidin pull-down and western blot for BMPR2 using the Clone 18 anti-BMPR2 antibody. The 130-145 kDa wild type BMPR2 product was detected in the streptavidin pull-down in control and F108 cultured lymphocytes, but the 120 kDa BMPR2ΔEx2 band was not (Figure 1D, right panel). These data indicate that the BMPR2ΔEx2 mutant product resulting from the in-frame deletion of *BMPR2 EXON2* in the F108 HPAH patient does not correctly traffic to the cell surface in cultured lymphocytes. 

### Characterization of the Bmpr2ΔEx2 product in pulmonary endothelial cells from Bmpr2^ΔEx2/+^ mice

**Figure 2:**
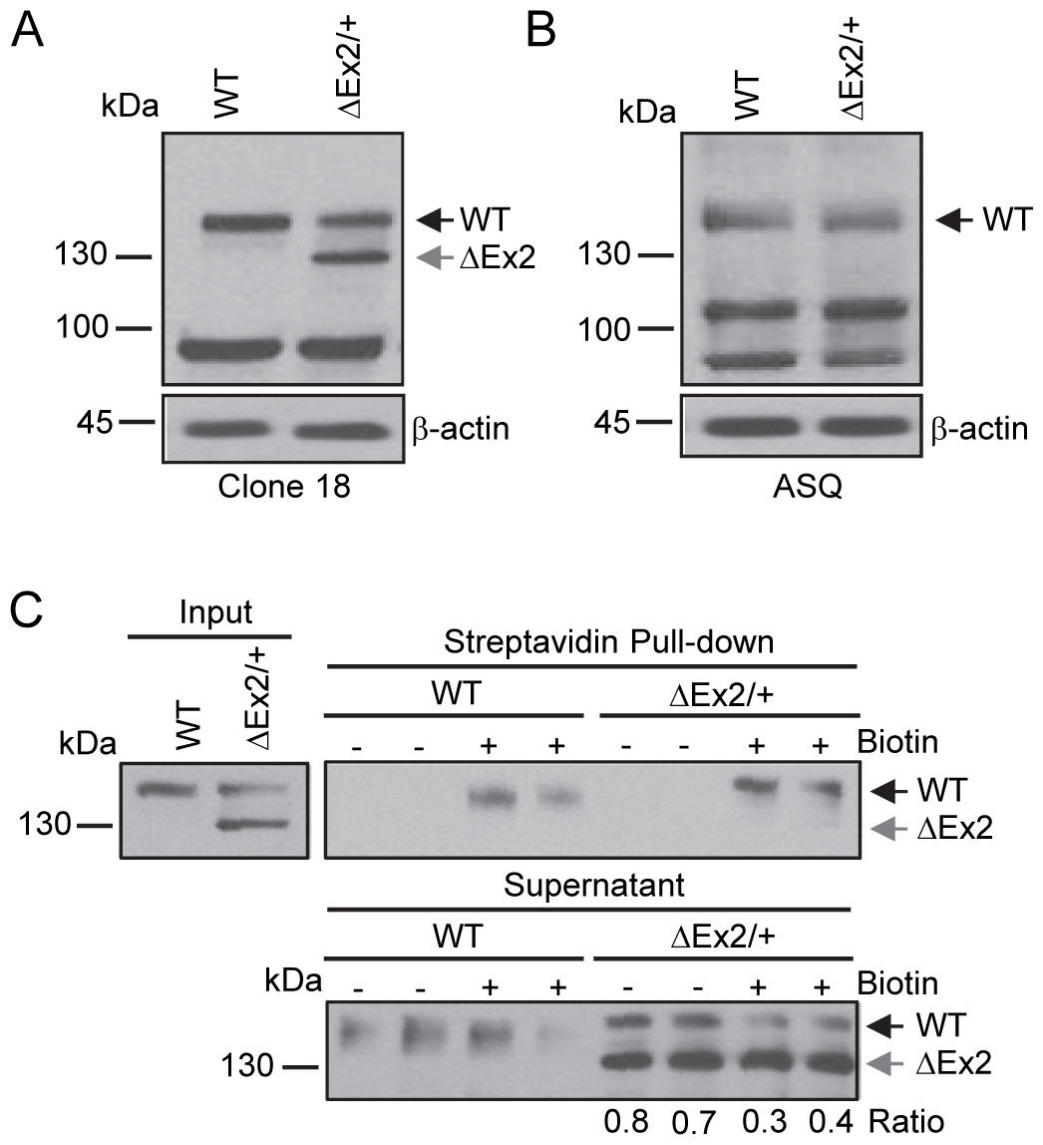
Characterization of the endogenously expressed *Bmpr2* mutant product in pulmonary endothelial cells from *Bmpr2* ^*ΔEx2/+*^ mice. Studies were performed using conditionally immortalized PECs (ciPECs) isolated from wild type control and *Bmpr2* ^ΔEx2/+^ mice and replicated at least three times. A, Western blot using the Clone 18 anti-BMPR2 antibody in wild type (WT) and *Bmpr2* ^ΔEx2/+^ (ΔEx2/+) ciPEC lysates. Both cell lines expressed a 150 kDa wild type Bmpr2 product (WT). *Bmpr2* ^*ΔEx2/+*^ ciPECs also expressed a 130 kDa product (ΔEx2). B, Western blot using ASQ anti-BMPR2 antibody. Wild type control and *Bmpr2* ^*ΔEx2/+*^ ciPECs expressed 150 kDa WT Bmpr2, but the 130 kDa product was not detected. C, Cell surface expression of Bmpr2 in ciPECs. ciPECs were labeled with membrane impermeable biotin and cell surface expression of Bmpr2 detected in streptavidin pull-down of cell lysates. Anti-BMPR2 Clone 18 antibody detected the 150 kDa wild type Bmpr2 in control and *Bmpr2* ^*ΔEx2/+*^ ciPECs after streptavidin pull-down but not the 130kDa Bmpr2ΔEx2 mutant product. Lower panel, Western blot for Bmpr2 in the supernatant remaining after depletion of cell surface proteins by streptavidin pull-down. The ratios of 150 kDa WT Bmpr2 and the 130 kDa Bmpr2ΔEx2 mutant band intensities in *Bmpr2* ^*ΔEx2/+*^ ciPECs supernatants after depletion of cell surface proteins are indicated below the lower panel.

To evaluate expression and trafficking of the same, endogenously expressed, BMPR2ΔEx2 mutant product in a more physiologically relevant cell type, we isolated conditionally immortalized pulmonary endothelial cells (ciPECs) from *Bmpr2* ^*ΔEx2/+*^ mice. *Bmpr2* ^*ΔEx2/+*^ mice carry the same, heterozygous in-frame deletion of *Exon 2* found in F108 HPAH patients [26]. The anti-BMPR2 antibody Clone 18 detected a 150 kDa band in control (wild type) and *Bmpr2* ^*ΔEx2/+*^ ciPECs, but an additional 130 kDa band was only detected in the *Bmpr2* ^*ΔEx2/+*^ ciPECs (Figure 2A). To determine if the 130 kDa band was the predicted product resulting from an in-frame deletion of *Bmpr2 Exon2*, we performed western blot analysis using the anti-BMPR2 antibody ASQ, raised against the peptide sequence in Exon2 (Figure 1A). The wild type 150kDa Bmpr2 band was detected in both the control and *Bmpr2* ^*ΔEx2/+*^ ciPECs, but the 130kDa product was not detectable in the *Bmpr2* ^*ΔEx2/+*^ciPECs using this antibody (Figure 2B). These data indicate that *Bmpr2* ^ΔEx2/+^ ciPECs expressed high levels of the Bmpr2ΔEx2 mutant product, which is detected as a 130 kDa band on western blot and is distinct from the 150 kDa wild type Bmpr2 product. 

We performed cell surface biotinylation studies to determine whether the BmprΔEx2 mutant product was expressed at the cell surface of *Bmpr2* ^*ΔEx2/+*^ciPECs. The 150 kDa wild type product was detected in the streptavidin pull-down in both control and *Bmpr2* ^ΔEx2/+^ ciPECs (Figure 2C). In contrast, the 130 kDa Bmpr2ΔEx2 mutant product was not detected in the streptavidin pull-down. Additionally, while there was reduced expression of the wild type Bmpr2 protein in control and *Bmpr2* ^ΔEx2/+^ ciPECs in the supernatant remaining after depletion of cell surface biotinylated proteins by streptavidin pull-down, expression of the 130kDa Bmpr2ΔEx2 mutant product was not reduced (Figure 2C, lower panel). These data indicate that like the 120 kDa BMPR2ΔEx2 product in F108 HPAH patient cultured lymphocytes, the 130 kDa Bmpr2ΔEx2 mutant product does not traffic to the cell surface in *Bmpr2* ^ΔEx2/+^ ciPECs. 

### Differential glycosidase sensitivity of the Bmpr2ΔEx mutant product

**Figure 3:**
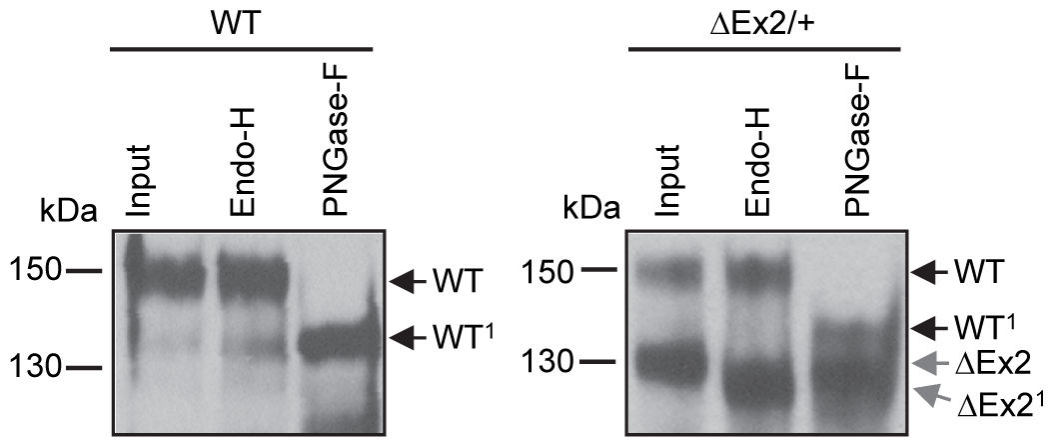
Differential N-linked glycosidase sensitivity of wild type Bmpr2 and Bmpr2ΔEx2 mutant products. Wild type control and *Bmpr2* ^*ΔEx2/+*^ ciPEC lysates were immunoprecipitated and digested with N-linked glycosidases Endo-H or PNGase-F. The 150 kDa wild type Bmpr2 bands in both control and *Bmpr2* ^*ΔEx2/+*^ ciPECs were sensitive to PNGase-F digestion, determined by a 15 kDa mobility shift (WT>WT^1^), but were insensitive to Endo-H digestion. Unlike wild type Bmpr2, the 130 kDa Bmpr2ΔEx2 mutant product in *Bmpr2* ^*ΔEx2/+*^ ciPECs was sensitive to Endo-H digestion (ΔEx2>ΔEx2^1^).

We used N-linked glycosidases to determine if the Bmpr2ΔEx2 mutant product was able to traffic correctly through the endoplasmic reticulum (ER) and Golgi apparatus. N-linked glycosylated proteins that are processed in the ER are sensitive to Endo-H glycosidase, but become resistant to Endo-H once the glycoprotein has passed through to the trans-Golgi [36]. In contrast, PNGase-F de-glycosylates all N-linked glycosylated proteins irrespective of their maturation state. This sensitivity to glycosidase digestion can be detected by a change in protein mobility resulting from cleavage of glycan moieties. As expected, the 150 kDa wild type Bmpr2 product in control and *Bmpr2* ^*ΔEx2/+*^ ciPECs underwent a mobility shift after treatment with PNGase-F, but was resistant to Endo-H digestion (Figure 3). In contrast, the 130 kDa Bmpr2ΔEx2 mutant product underwent a mobility shift after digestion with PNGase-F and Endo-H (ΔEx2>ΔEx2^1^). This indicates that wild type Bmpr2 is correctly processed through the ER and Golgi but that the 130 kDa Bmpr2ΔEx2 mutant product is likely to be retained in the ER. 

### Chemical chaperones restore trafficking of the Bmpr2ΔEx2 mutant product to the cell surface

**Figure 4:**
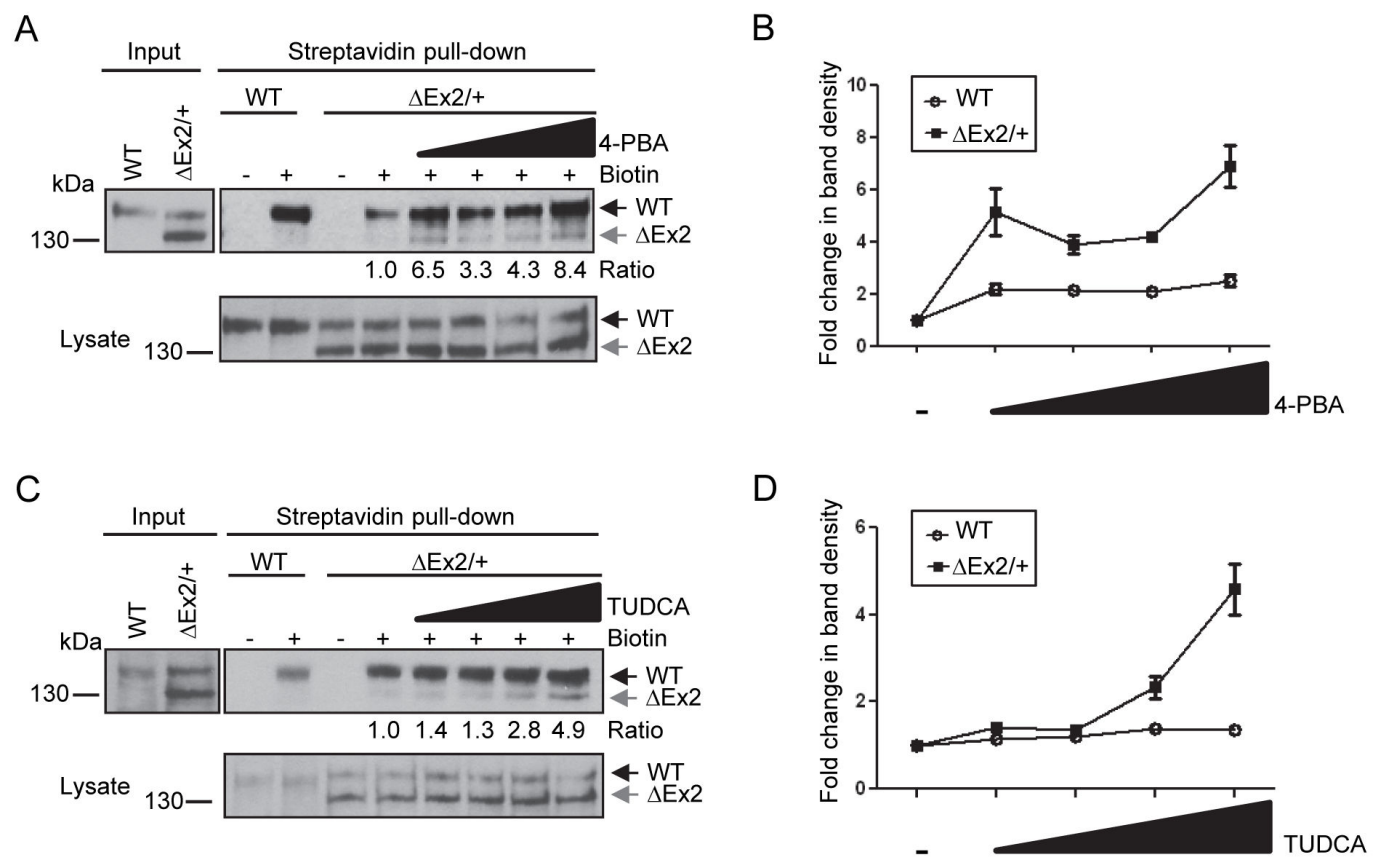
Chemical chaperones partially restore Bmpr2ΔEx2 mutant product expression at the cell surface. A, Cell surface expression of Bmpr2ΔEx2 in *Bmpr2* ^*ΔEx2/+*^ ciPECs treated with 4-PBA. *Bmpr2* ^*ΔEx2/+*^ ciPECs were treated for 48 hours with 100µM, 250µM, 500µM or 1mM of 4-PBA. Monolayers were then labeled with membrane impermeable biotin and biotinylated cell surface proteins pulled-down with streptavidin agarose beads. Western Blot was performed with Clone 18 anti-BMPR2 antibody. The 150 kDa wild type Bmpr2 product was detected in the streptavidin pull-down in control and *Bmpr2* ^ΔEx2/+^ ciPECs, but the 130 kDa Bmpr2ΔEx2 mutant product was not detected. After treating with the chemical chaperone 4-PBA, the 130kDa Bmpr2ΔEx2 mutant product was detected and there was increased expression of the wild type Bmpr2 product in the streptavidin pull-down. Numbers shown below the upper panel indicate the ratio of the 130 kDa Bmpr2ΔEx2 band before and after treatment with 4-PBA. Lower panel, expression of wild type Bmpr2 and Bmpr2ΔEx2 in ciPEC cell lysates with 4-PBA treatment. B, Quantification of wild type Bmpr2 and Bmpr2ΔEx2 band densities after 4-PBA treatment relative to untreated controls from three independent experiments, standard error is indicated. C, Cell surface expression of Bmpr2ΔEx2 in *Bmpr2* ^*ΔEx2/+*^ ciPECs treated with TUDCA. Wild type and *Bmpr2* ^*ΔEx2/+*^ ciPECs were treated for 5 hours with 50µM 100µM, 250µM or 500µM of TUDCA. Streptavidin pull-down shows that the 130kDa Bmpr2ΔEx2 mutant product was partially restored at the cell surface and there was a slight increase in wild type Bmpr2 with TUDCA treatment (1.4 fold increase with 500µM TUDCA versus untreated cells). Numbers shown below the upper panel indicate the ratio of the 130 kDa Bmpr2ΔEx2 band before and after treatment with TUDCA Lower panel, expression of wild type Bmpr2 and Bmpr2ΔEx2 in ciPEC cell lysates with TUDCA treatment. D, Quantification of wild type Bmpr2 and Bmpr2ΔEx2 band densities after TUDCA treatment relative to untreated controls from three independent experiments.

Since the retention of mutant protein in the ER is often a consequence of incorrect protein folding [37], we wanted to evaluate the effects of two chemical chaperones known to aid in protein folding, 4-PBA and TUDCA [24,25,38-43] , on trafficking of the Bmpr2ΔEx2 mutant product to the cell surface. *Bmpr2* ^*ΔEx2/+*^ ciPECs treated with increasing concentrations of 4-PBA show a dose-dependent increase expression of the 130kDa Bmpr2ΔEx2 mutant product in the streptavidin pull-down (Figure 4A/B). Treatment with 4-PBA also increased the cell surface expression of the 150 kDa wild type Bmpr2 protein in *Bmpr2* ^*ΔEx2/+*^ ciPECs, but to a relatively lesser extent than Bmpr2ΔEx2. An aliquot of cell lysates taken before the streptavidin pull-down showed no change in expression of wild type Bmpr2 or Bmpr2ΔEx2 with the addition of 4-PBA (Figure 4A, lower panel). An additional chemical chaperone, TUDCA added to cells prior to biotinylation also increased cell surface expression of the 130kDa Bmpr2ΔEx2 mutant protein in *Bmpr2* ^ΔEx2/+^ ciPECs (Figure 4C/D). There was also a small increase in cell surface expression of wild type Bmpr2 after treatment with TUDCA (a 1.4 fold increase with 500µM TUDCA versus untreated cells). These data indicate that chemical chaperones increase cell surface expression of Bmpr2ΔEx2 in *Bmpr2* ^ΔEx2/+^ ciPECs, and suggest that ER retention of Bmpr2ΔEx2 results from mis-folding of the mutant protein. 

### BMP signaling defects in primary pulmonary endothelial cells from Bmpr2^ΔEx/+^ mice

**Figure 5:**
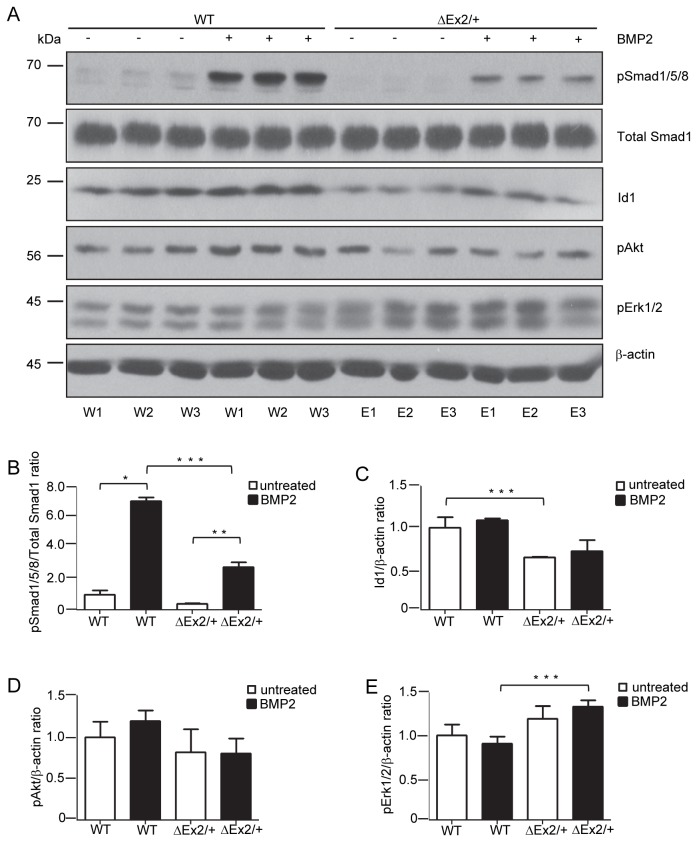
BMP signaling defects in primary pulmonary endothelial cells from *Bmpr2* ^ΔEx2/+^ mice. Primary PECs were isolated from 3 different wild type control (WT) and 3 *Bmpr2* ^*ΔEx2/+*^ mice (ΔEx2). A, Western blot of cell lysates isolated from wild type control (W1-W3) and *Bmpr2* ^*ΔEx2/+*^ (E1-E3) PECs treated with 10ng/ml BMP2 for 4 hours. Antibodies indicated on the right. Densitometry for phospho-Smad1/5/8 (B), Id1 (C), pERK1/2 (D) and pAKT bands (E). Results expressed as mean +/- SEM, 3 per group. One-way ANOVA with post hoc Bonferroni correction, p<0.05 *WT +/-BMP2; **ΔEx2+/-BMP2; ***WT vs. ΔEx2. Experiment and isolation of primary PECs was replicated four times.

To determine the functional effects of the *Bmpr2* ^ΔEx2/+^ mutation on BMP-mediated signaling responses, we evaluated BMP signaling in early passage primary pulmonary endothelial cells (PECs). For this, six separate PEC isolates were prepared from 3 wild type control and 3 *Bmpr2* ^*ΔEx2/+*^ mice. There was a significant decrease in BMP-stimulated phospho-Smad1/5/8 expression in *Bmpr2* ^*ΔEx2/+*^ PECs associated with reduced basal and BMP-induced Id1 expression (Fig. 5A/B/C). There was also an increase in phospho-Erk1/2 expression in *Bmpr2* ^ΔEx2/+^ PECs (Figure 5A/D) but no significant change in phospho-Akt (Figure 5A/E). These studies indicate that *Bmpr2* ^ΔEx2/+^ PECs exhibit BMP signaling defects. 

### Chemical Chaperone 4-PBA restores BMP signaling defects in Bmpr2^ΔEx2/+^ PECs

**Figure 6:**
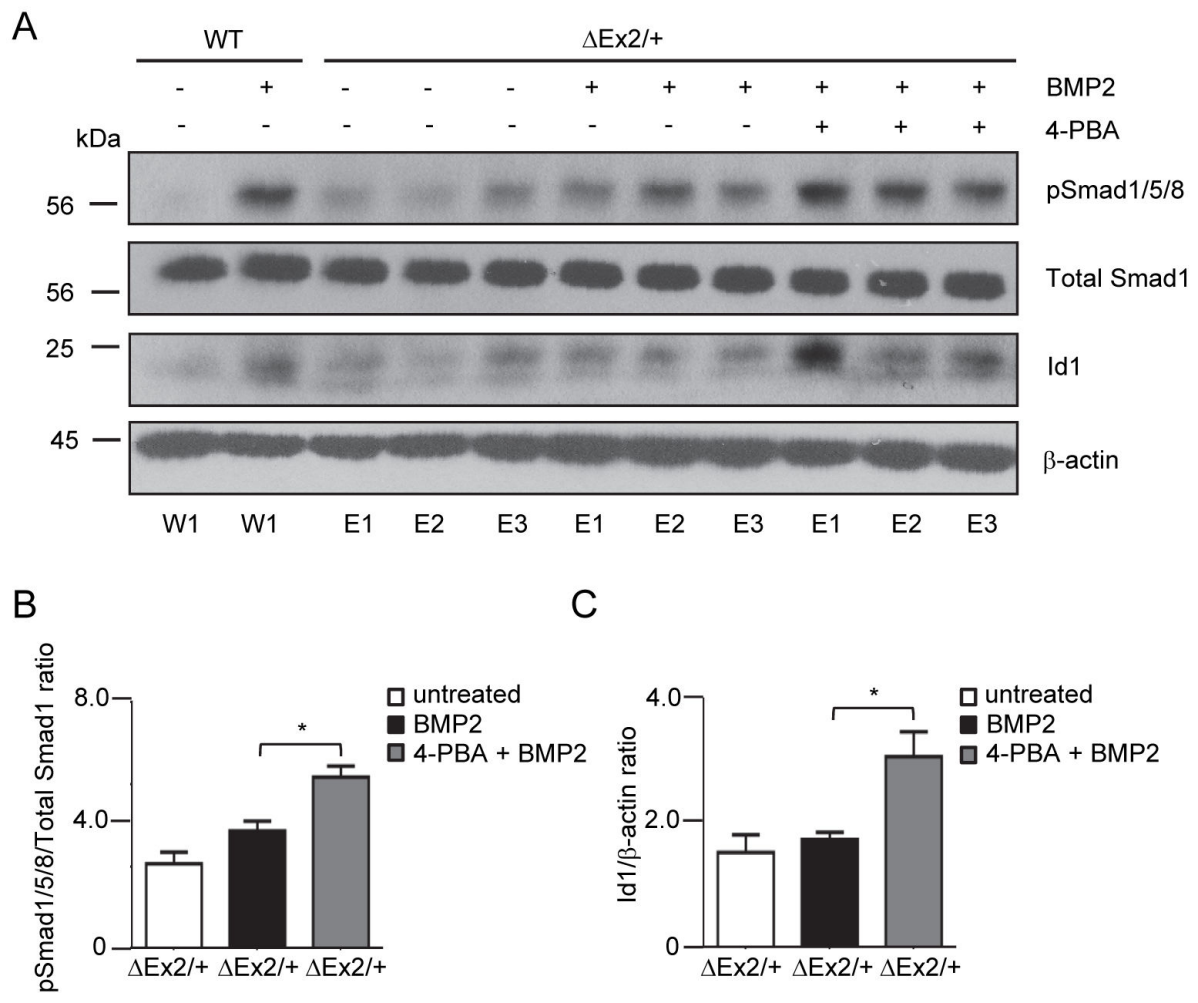
Treatment with 4-PBA rescues signaling defects in *Bmpr2* ^*ΔEx2/+*^ pulmonary endothelial cells. Individual primary PECs isolates obtained from 1 wild type control and 3 *Bmpr2* ^*ΔEx2/+*^ mice were treated with 1mM 4-PBA for 48 hours followed by BMP2 treatment for 4 hours, as indicated. A, Western blot for phospho-Smad1/5/8 and Id1 shows 4-PBA restores phospho-Smad1/5/8 and Id1 expression in *Bmpr2* ^*ΔEx2/+*^ PECs to expression levels similar to BMP-stimulated WT PECs. B, Densitometry for phospho-Smad1/5/8 and C, Id1 expressed as ratio to total Smad1 and β-actin respectively. Results expressed as mean +/- SEM of three individual PEC isolates per group. One-way ANOVA with post hoc Bonferroni correction, p<0.05 * ΔEx2/+ +BMP2 vs. ΔEx2/+ +BMP2+4-PBA.

To determine whether restoration of cell surface expression of Bmpr2ΔEx2 restores defective BMP signaling in *Bmpr2* ^*ΔEx2/+*^ PECs, we evaluated BMP signaling primary PECs isolates obtained from 1 wild type control and 3 *Bmpr2* ^*ΔEx2/+*^ mice treated with 4-PBA (Figure 6A). 4-PBA restored BMP2 induced Smad1/5/8 phosphorylation (Figure 6B) and BMP-induced Id1 protein expression in *Bmpr2* ^*ΔEx2/+*^ PECs to a level comparable to that seen in wild type PECs treated with BMP2 (Figure 6C). These data indicate that treatment with the chemical chaperone 4-PBA restores the defective BMP-Smad/Id1 signaling axis in *Bmpr2* ^*ΔEx2/+*^ PECs. 

## Discussion

In these studies, we provide the first evidence that an NMD negative *BMPR2* mutation found in patients with HPAH is expressed endogenously, and that this mutant protein product is mis-folded and incorrectly trafficked to the cell surface. We also show that chemical chaperones partially restore cell surface expression of the same *Bmpr2* mutant product in pulmonary endothelial cells from mice carrying the same heterozygous germ line mutation, and that treatment with chemical chaperones rescues the associated BMP signaling defects in these cells. These data provide the first evidence for the therapeutic use of chemical chaperones to correct endogenous signaling defects resulting from mis-folded *BMPR2* mutant products in patients with HPAH. 

Our studies focus on the expression and trafficking of these *BMPR2* mutant products in pulmonary endothelial cells from *Bmpr2* ^*ΔEx2/+*^ mice since BMPR2 is highly expressed in endothelial cells in the pulmonary vasculature, and endothelial expression of BMPR2 has been implicated in maintaining normal pulmonary vasculature tone and integrity [8,11,13,14,44,45]. We have also previously shown that mice with heterozygous in-frame deletion of *Bmpr2 Exon2* (*Bmpr2* ^*ΔEx2/+*^) have reduced endothelium-dependent vasodilator responses in the pulmonary vasculature [8], and approximately 30% of mice with conditional ablation of *Bmpr2* in endothelial cells develop spontaneous pulmonary hypertension [11]. These findings suggest that endothelial BMPR2 plays an important role in the regulating pulmonary vascular function, and suggests strategies to correct BMP signaling in pulmonary endothelial cells may have beneficial effects on the pulmonary vasculature in mice and HPAH patients with *BMPR2* mutations. 

We found that the Bmpr2ΔEx2 mutant product was expressed at high levels in pulmonary endothelial cells isolated from *Bmpr2* ^ΔEx2/+^ mice. The identity of this band was verified as the in-frame deletion of Exon2 by using an antibody raised against the peptide sequence encoded by *BMPR2 Exon2* (ASQ). This in-frame deletion of Exon2 is also expressed in the F108 HPAH patient-derived lymphocytes. However, there are differences in mobility of both wild type BMPR2 and the BMPR2ΔEx2 products in mouse ciPECs and cultured human lymphocytes. This is likely due to cell-type dependent differences in post-translational modifications/glycosylation of BMPR2. In addition, unlike F108 HPAH patient–derived lymphocytes, the presumed Bmpr2ΔEx2 mutant product in *Bmpr2* ^*ΔEx2/+*^ ciPECs had an expression level similar to the 150 kDa wild type Bmpr2 product. This indicates that there are cell-type dependent differences in expression of the mutant allelic products, and that high levels of Bmpr2ΔEx2 expression in endothelial cells may exert more profound effects on cellular function than in different cell types. Cell surface biotinylation and N-glycosidase sensitivity assays indicate that the Bmpr2ΔEx2 mutant product does not traffic correctly to the cell surface and is retained in the ER. Additionally, chemical chaperones 4-PBA and TUDCA, agents that are known to aid in protein folding [24,25,38-43], partially restore trafficking of the Bmpr2ΔEx2 mutant product to the cell surface. These findings suggest that the Bmpr2ΔEx2 is retained in the ER as a result of mis-folding of the mutant product. Since deletion of *Exon2* in Bmpr2ΔEx2 results in a loss of 3/10 cysteine residues located in the ligand-binding domain of Bmpr2, it is likely that mis-folding occurs as a result of a breakdown in paired cysteine disulfide bond formation which is required for correct Bmpr2 structure and folding in the ER [46-49]. 

Our data suggest that chemical chaperones may be used to correct BMP signaling defects in HPAH patients with NMD negative *BMPR2* mutations. Functional studies show that 4-PBA treatment can restore CFTR receptor ΔF508 mutant trafficking and function in cells derived from Cystic Fibrosis (CF) patients [25], and 4-PBA has recently been used in clinical trials as a chemical chaperone to treat CF [50,51]. Treatment with chemical chaperones like 4-PBA aid in protein folding and trafficking to the cell surface; however it is important to note that there are a number of NMD negative *BMPR2* mutations located in regions of the protein that would not benefit from trafficking to the cell surface. For example, mutations located in the kinase domain or cytoplasmic tail domains of BMPR2 in particular may not benefit from chaperone treatment. For this strategy to work in HPAH patients with NMD negative *BMPR2* mutations, the mutations must allow at least partial BMPR2 function when the mutant product is expressed at the cell surface. Correcting a protein-folding defect of an HPAH NMD negative *BMPR2* mutant product that has dominant negative activity when expressed at the cell surface might have adverse effects on BMP signaling. This has been demonstrated with the kinase inactive HPAH *BMPR2 C483R* mutation in in a heterologous over-expression system [23], but still needs to be evaluated endogenously in HPAH patient-derived cells. In the case of Bmpr2ΔEx2, correction of BMP signaling defects by chemical chaperones may be occurring through two non-mutually exclusive mechanisms. While the Bmpr2ΔEx2 mutation interferes with the structure of the ligand-binding domain of the receptor (and presumably therefore interferes with ligand binding to the receptor), Bmpr2ΔEx2 may still participate in functional hetero-tetrameric complexes with wild type BMPR2 and other BMP Type 1 receptors that can independently directly engage BMP ligands at the cell surface. Interestingly, 11% of NMD negative *BMPR2* mutations are in the ligand-binding domain of BMPR2 [18], and may similarly benefit from corrected trafficking to the cell surface. However, in addition to restoring cell surface expression of the mutant allelic product, our data show that 4-PBA, and to a lesser extent TUDCA, increase cell surface expression of wild type Bmpr2 in *Bmpr2* ^ΔEx2/+^ ciPECs. Since mis-folding of Bmpr2ΔEx2 may trap some wild type Bmpr2 in the ER, it is possible that this effect is a direct consequence of 4-PBA-dependent correction of the cell surface trafficking defect of the mutant allele. Given that expression levels of Bmpr2 have been shown to be important in maintaining normal signaling function [2,35], this increase in expression of wild type Bmpr2 protein at the cell surface may account for the restoration in BMP signaling in *Bmpr2* ^ΔEx2/+^ ciPECs after treatment with chemical chaperones. However, irrespective of the mechanisms restoring BMP signaling in *Bmpr2* ^ΔEx2/+^ ciPECs, our data indicate that there may be a subset of HPAH patients with NMD negative *BMPR2* mutations that show beneficial responses to protein folding agents. Further analysis of endogenous *BMPR2* mutant product folding and signaling defects in HPAH patients carrying different NMD negative *BMPR2* mutations will have to be performed to determine which patients might benefit from this therapy.

Our studies provide the first evidence that chemical chaperones can be used to rescue BMP signaling defects associated with endogenously expressed NMD negative HPAH *BMPR2* mutations in the pulmonary endothelium. Additionally, the chemical chaperones we evaluated, 4-PBA and TUDCA, are FDA-approved drugs and commercially available supplements, respectively, and are in clinical trials for other diseases caused by mis-folded proteins [50-52]. Therefore, while further analysis of endogenous *BMPR2* mutant product folding and signaling defects in HPAH patients will have to be performed to determine which patients might benefit from this therapy, our data suggest an additional disease modifying therapy that may benefit a subset of HPAH patients with NMD negative *BMPR2* mutations. 
